# Hints for the Profession

**Published:** 1891-08

**Authors:** A. V. Banes

**Affiliations:** St. Joseph, Mo.


### Hints for the Profession.

BY A. V. BANES, M.D., ST. JOSEPH, MO.

The major portion of our lives is passed in the office, more especially those whose who enjoy a large city practice. How very important it is that we should have everything neat, clean and pleasant. Our patients will wait more willingly if they have a few pictures to look at, a small reservoir containing a few gold fish, and a few ornaments or little brackets might adorn the walls. Our failure or success in a business way depends more on our surroundings than most of us are aware. Intelligent and cultured people look to the environments on entering an office when calling for the first time, and your ability as a flourishing and successful physician will be very correctly guaged by their first impressions. A bright, cosy, attractive office will do much towards making your patients satisfied and contented while awaiting their turn to consult you; and they will look over the daily papers and the few periodicals they may find on the table with a great deal of pleasure. Do not fear that money expended in this way will not be returned a thousand fold. You never see a cheerful, well-appointed office but you find a wide open doctor, well equipped to practice his special branches successfully. You will certainly attract a better class of patrons who are amply able and willing to pay the price of your services.

If possible, have your office and residence together, and then your wife can superintend, and see that your rooms are tidy and clean. Have a bright and intelligent office boy to make appointments and look after the door during your hours; and discard the time-worn slate with the old chestnut, "Return soon." Have your wife keep your books, and give her your calls morning, noon and night. She will make you rich if you trust her.

Keep your private office books yourself. Collect your bills every thirty or sixty days, or take notes every third or fourth month. I find by taking notes and allowing the banks to collect it answers well, as people will pay an institution more promptly than a doctor.

